# Role of the ion channel, transient receptor potential cation channel subfamily V member 1 (TRPV1), in allergic asthma

**DOI:** 10.1186/s12931-016-0384-x

**Published:** 2016-06-02

**Authors:** Katie Baker, Kristof Raemdonck, Bilel Dekkak, Robert J. Snelgrove, John Ford, Fisnik Shala, Maria G. Belvisi, Mark A. Birrell

**Affiliations:** Respiratory Pharmacology, Airway Disease Section, National Heart and Lung Institute, Faculty of Medicine, Imperial College London, Exhibition Road, London, SW7 2AZ UK; Department of Anatomy, Faculty of Medicine, University of Porto, Alameda Prof. HernâniMonteiro, 4200-319 Porto, Portugal; Center for Health Technology and Services Research (CINTESIS), Faculty of Medicine, University of Porto, Rua Dr. Plácido da Costa, 4200-450 Porto, Portugal; Leukocyte Biology, Imperial College, London, UK; ArioPharma Limited, Iconix Park, London Road, Pampisford, CB22 3EG UK; Asthma UK Centre in Allergic Mechanisms of Asthma, Imperial College London, London, UK

**Keywords:** Lung, Asthma, Ion channel, Inflammation

## Abstract

**Background:**

Asthma prevalence has increased world-wide especially in children; thus there is a need to develop new therapies that are safe and effective especially for patients with severe/refractory asthma. CD4^+^ T cells are thought to play a central role in disease pathogenesis and associated symptoms. Recently, TRPV1 has been demonstrated to regulate the activation and inflammatory properties of CD4^+^ cells. The aim of these experiments was to demonstrate the importance of CD4^+^ T cells and the role of TRPV1 in an asthma model using a clinically ready TRPV1 inhibitor (XEN-D0501) and genetically modified (GM) animals.

**Methods:**

Mice (wild type, CD4 ^−/−^ or TRPV1 ^−/−^) and rats were sensitised with antigen (HDM or OVA) and subsequently topically challenged with the same antigen. Key features associated with an allergic asthma type phenotype were measured: lung function (airway hyperreactivity [AHR] and late asthmatic response [LAR]), allergic status (IgE levels) and airway inflammation.

**Results:**

CD4^+^ T cells play a central role in both disease model systems with all the asthma-like features attenuated. Targeting TRPV1 using either GM mice or a pharmacological inhibitor tended to decrease IgE levels, airway inflammation and lung function changes.

**Conclusion:**

Our data suggests the involvement of TRPV1 in allergic asthma and thus we feel this target merits further investigation.

## Background

Asthma is an inflammatory airway disease characterised by variable expiratory flow limitation and is associated with respiratory symptoms, such as wheeze, shortness of breath, chest tightness and cough [1]. Asthma prevalence has increased worldwide. It is estimated that it affects approximately 300 million people and it is believed that over 100 million more people will be affected by 2025 [2]. Therefore, there is a need to develop new therapies that are safe and effective, especially for patients with severe/refractory asthma. CD4^+^ T cells are thought to play a central role in the disease pathogenesis and associated symptoms [3, 4]. Recently, it has been shown that the ion channel, transient receptor potential cation channel subfamily V member1 (TRPV1) is present on T cells [5] and it regulates the activation and inflammatory properties of CD4^+^ cells [6, 7].

TRPV1 is a non-selective cation channel and is a member of a large family of TRP ion channels. It can be activated by a diverse range of endogenous and exogenous chemical ligands, low pH and high temperatures [8]. In addition to the recent data demonstrating a role in CD4^+^ T cell function, previous reports have indicated an association with asthma. For example, TRPV1 polymorphism has been associated with childhood asthma [9] and a loss of function. The TRPV1 genetic variant has been shown to be associated with a lower risk of childhood asthma or the presence of wheezing [10]. In another study, TRPV1 gene expression and Th1/Th2 cytokines were found to be increased compared to controls and associated with childhood onset asthma [11]. Furthermore, inflammatory stimuli have been reported to increase the expression of TRPV1 [12] and other studies have suggested an increase in TRPV1 expression in asthmatic patients [13] and also in animal models of asthma [14]. Based on this body of data, we, and others, have hypothesised that TRPV1 plays a role in allergic asthma [15–17]. The aim of this study was to determine the role of TRPV1 in two distinct models of allergic asthma by adopting two different approaches; incorporating the use of genetically modified TRPV1^−/−^ animals and utilising a selective, potent, clinically ready, small molecule TRPV1 inhibitor [18] to test the hypothesis.

## Methods

### Animals

Male and female C57BL/6 mice (16–20 g) were originally obtained from Harlan UK Limited (Bicester, UK). Male CD4^−/−^ mice were obtained via the Swiss Immunological Mouse Repository. TRPV1^−/−^ mice were purchased from Jackson Labs.TRPA1^−/−^ mice were obtained from a generous donation from Prof. David Julius (University of California) via Prof Peter Zygmunt (Lund University). All of the genetically modified (GM) lines were on a C57BL/6 background and colonies of sufficient size, including the wild type (WT) controls, were established *in house*. Age matched male mice were used for the studies.Male Brown Norway rats (175-225gm) were purchased from Charles River, Germany, and housed for at least 5 days before beginning treatments with food and water supplied ad libitum*.* All protocols were approved by a local ethical review process (Animal Welfare and Ethical Review Body) and strictly adhered to the Animals (Scientific Procedures) Act 1986 UK Home Office guidelines. The in vivo work was performed under a project licence (PPL70/7212) by staff holding personal licences that were trained in the relevant techniques and according to the ARRIVE guidelines [19].

### Compounds and materials

XEN-D0501 was a gift from Dr J. Ford at ArioPharma Ltd (Unit 3, Iconix Park, Pampisford, Cambs, CB22 3EG). He also provided the pharmacokinetic data to guide dose selection (along with internally generated pharmacodynamic data [18]). Reagents were purchased from Sigma-Aldrich (Poole, UK) unless otherwise described.

### Confirmation of phenotype/genotype of the GM lines

While establishing the colony, the phenotype of the CD4^−/−^mice was confirmed by assessing cell types in the lung. Wild type (WT) and CD4^−/−^ male mice (18–22 gm) were culled with an overdose of pentobarbitone (200 mg/kg, i.p.). The blood was removed from the lung vessels by perfusing with normal saline prior to harvesting. The tissue was then cleaned, chopped and the cells collected via an enzymatic digestion based on a method described previously [20]. The numbers of CD4^+^ cells, CD8^+^ T cells, CD19^+^ cells (B cells), eosinophils, neutrophils and alveolar macrophages were determined by flow cytometry. Lung mast cell populations were determined by Toluidine blue histological analysis (see below). The genetic status of the TRP knockout lines was confirmed using a standard genotyping procedure.

### Flow cytometry

Single-cell suspensions were stained for surface markers in PBS containing 0.1 % sodium azide and 1 % BSA for 30 min at 4 °C and fixed with 2 % paraformaldehyde. Data was acquired on a BD FACS Fortessa machine (BD Biosystems, UK). Forward scatter and side scatter gates were used to exclude debris and dead cells were excluded using a fixable near IR dead cell stain kit for 633 or 635 nm excitation. Cell types were characterised by their forward and side scatter profiles and by their phenotypes (Table 1).

**Table 1:** Characterisation of immune cells by flow cytometry

Cell Type	Surface Marker Phenotype	Monoclonal Antibody Conjugate	Catalogue Number	Dilution
B Cells	CD19^+^	CD19-FITC (BD Biosciences)	557398	1/100
	CD3^−^	CD3-PECy7 (eBioscience)	25–0031	1/200
Natural Killer (NK) Cells	NKp46^+^	NKp46-PE (eBioscience)	12–3351	1/200
	CD3^−^	CD3-PECy7 (eBioscience)	25–0031	1/200
CD4^+^ T Cells	CD4^+^	CD4-PerCP (BD Biosciences)	553052	1/200
	CD3^+^	CD3-PECy7 (eBioscience)	25–0031	1/200
CD8^+^ T Cells	CD8^+^	CD8-APC (BD Biosciences)	553035	1/200
	CD3^+^	CD3-PECy7 (eBioscience)	25–0031	1/200
Neutrophils	Ly-6G^high^	Ly6G-FITC (BD Biosciences)	551460	1/100
	CD11b^high^	CD11b-PerCP (eBioscience)	45–0112	1/400
	CD11c^low^	CD11c-APC (BD Biosciences)	550261	1/200
	F4/80^low^	F4/80-PE (eBioscience)	12–4801	1/50
Alveolar Macrophages	CD11b^low-int.^	CD11b-PerCP (eBioscience)	45–0112	1/400
	CD11c^high^	CD11c-APC (BD Biosciences)	550261	1/200
	F4/80^high^	F4/80-PE (eBioscience)	12–4801	1/50
Inflammatory monocytes/ macrophages	CD11b^high^	CD11b-PerCP (eBioscience)	45–0112	1/400
	CD11c^low^	CD11c-APC (BD Biosciences)	550261	1/200
	F4/80^high^	F4/80-PE (eBioscience)	12–4801	1/50
Eosinophils	CD11b^high^	CD11b-PerCP (eBioscience)	45–0112	1/400
	CD11c^low^	CD11c-APC (BD Biosciences)	550261	1/200
	SiglecF^high^	SiglecF-PE (BD Biosciences)	552126	1/200

### Mast cell enumeration

Mast cells were identified using a standard Toluidine blue histological stain. Mice were culled via overdose with Sodium Pentobarbitone and the systemic circulation perfused with saline. The trachea was then cannulated and the lungs perfused with formalin before being placed in formalin for 24 h. Following this, they were transferred into 70 % ethanol until paraffin wax embedding and slicing could take place. 4 μm sections were cut from the processed lung samples. The sections were stained using a standard toluidine blue staining protocol [21]. Briefly, the lung sections were dewaxed using Histochoice clearing agent ® (Sigma, UK) and rehydrated in a series of ethanol dilutions (100 %, 90 %, 70 %). The slices were then washed in deionised water and stained in 0.1 % Toluidine Blue (Sigma, UK) for 5mins. Sections were then washed in distilled water before the slices were dehydrated using a series of ethanol dilutions (70 %, 90 %, 100 %). The slices were left to dry at room temperature and mounted onto glass slides. The stained sections were analysed under light microscopy at ×40 magnification, the observer blinded to the specimen identities. The numbers of mast cells per slide (3 slides per lung sample) were counted.

### House Dust Mite driven allergic asthma mouse model

Male mice were sensitised with HDM (0.5 μg/kg – from Greer, USA, actual amounts of HDM) in saline (100 μl/mouse i.p.) on day 0 and 14. On days 24, 25 and 26 mice were challenged daily either with vehicle (saline, intranasally) or 1.25 μg/kg HDM (in 50 μl dose volume, intranasally) under light anaesthesia (inhaled isoflurane) as described previously [22]. 72 h after the final HDM challenge airway reactivity (AR) to inhaled 5-HT was assessed using whole body plethysmography (WBP; Penh). Previous work by our group, and others, have highlighted the important role airway sensory nerves play in respiratory disease [23], this is the reason lung function measurements are performed in conscious animals. One hour after AR assessment (to allow recovery from the bronchospasm) the mice were culled with an over dose of pentobarbitone (200 mg/kg, i.p.). Tissue was collected for genotyping. Heparinised blood samples were collected via cardiac puncture for plasma IgE levels. The lungs were lavaged via a tracheal cannula (3 times with 0.3 ml of RPMI, pooled) and total white cell number and differential percentage in the BAL fluid assessed (as described previously, [24]). The remaining lavage samples were kept at −80 °C. Total IgE levels were measured using BD OptEIATM set for mouse immunoglobulin E (BD Biosciences, Oxford, UK) in accordance with the manufacturer’s instructions.

### OVA-driven allergic asthma rat model

Male Brown-Norway rats (200–250 g) were sensitised on day 0, 14 and 21 with vehicle (saline with Alum 50:50, 1 ml/rat, i.p.) or OVA (100 μg/rat) administered with Alum (20 mg/rat aluminium hydroxide and 20 mg/rat magnesium hydroxide). All rats were challenged with OVA (1 % w/v, aerosolised for 30 min) on day 28 as previously described [23, 25]. Rats received vehicle (0.5 % MC + 0.2 % tween 80 in saline, 10 ml/kg, i.p., 2 sites) or TRPV1 inhibitor (XEN D0501, 10 mg/kg) 1 h before and 30 min after challenge. One hour after the antigen challenge the rats were placed in plethysmography chambers and Penh levels monitored over night. Following this the animals were given an overdose of pentobarbitone (200 mg/kg, i.p.) and a heparinised plasma sample collected via cardiac puncture. Bronchoalveolar lavage (BAL) was carried out (2 × 3 ml RPMI, 30 s each) and total/differential leukocyte numbers assessed.

OVA specific IgE levels were measured using ELISA. Briefly, 96 well plates were coated with OVA (20 ug/ml) and then blocked. Samples were added and left over night at room temperature. After washing Biotin anti-IgE was added for an hour. After washing anti-IgE was detected using horseradish peroxidase conjugated to streptavidin and visualised with tetramethylbenzidine substrate.

The level of airway inflammation was assessed on histologically prepared lung samples from the rat and mouse models using PAS (for mucus load) staining and inflammatory scoring. The scoring was performed by a trained technician blinded to the treatment groups. Full methods have been described previously [22].

### Data analysis and statistics

Data was expressed as mean ± S.E.M of n observations. A p value < 0.05 was taken as statistically significant and the actual statistical test employed indicated in the figure legends.

## Results

### Confirmation of phenotype/genotype of the GM lines

**Fig. 1:**
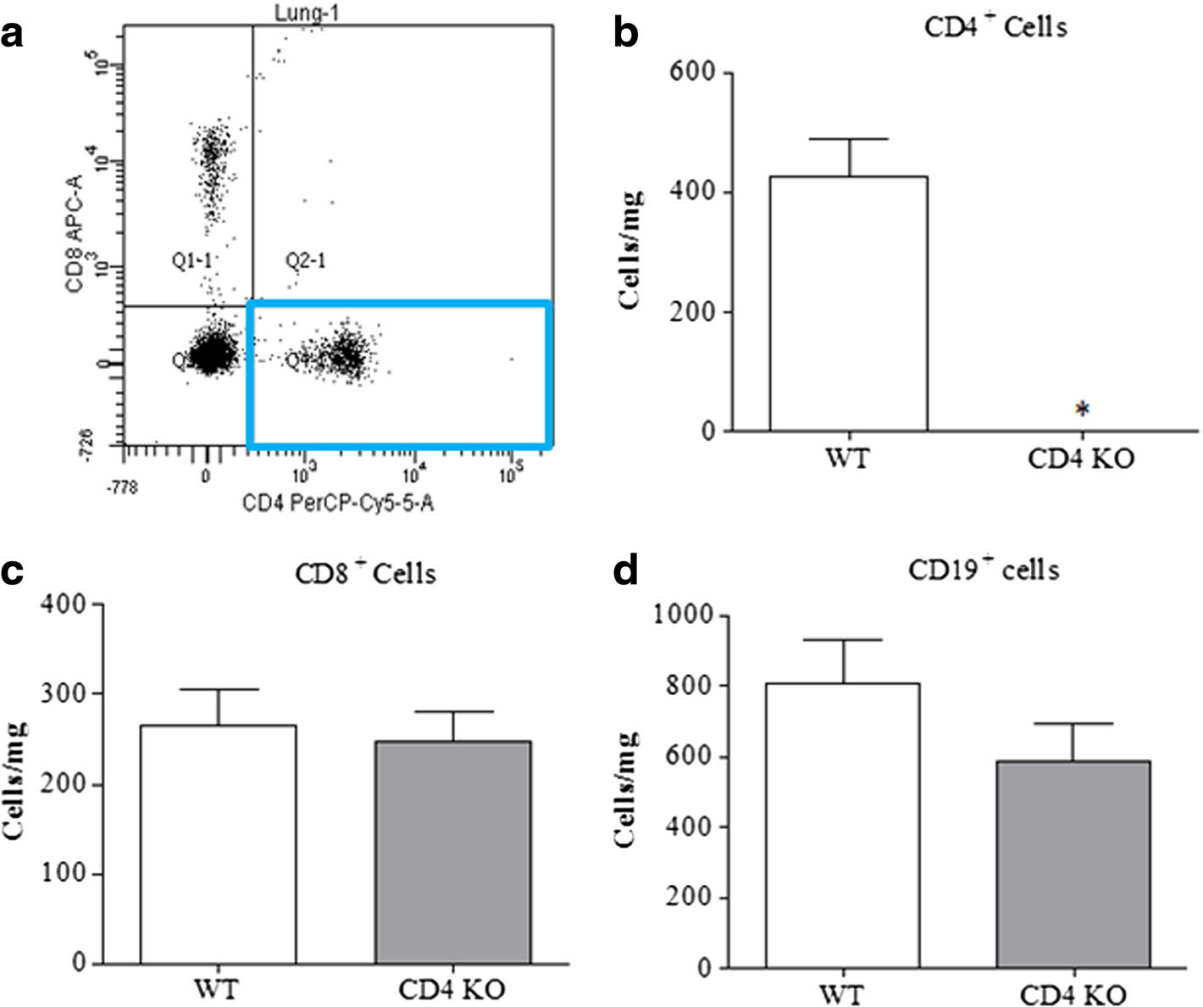
Characterising the CD4^+ −/−^ mice. **a** Representative scatter plot of CD4^+^and CD8^+^cells in the lungs of naive mice and the mean data for total CD4^+^ T cell numbers in wild type and CD4^+^ KO mice (*n* = 4, expressed at cells/mg of lung tissue) (**b**). **c** & **d**) show the mean numbers of CD8^+^ and CD19^+^ (B cells) cells, respectively. Data expressed as mean +/− s.e.m. * = *p* < 0.05 using students *T*-test (Mann-Whitney)

To confirm the phenotype of the CD4^+^ T cell KO mice and to investigate whether this impacted on other key allergic effector cells in the lung, we performed FACS analysis. Figure 1a shows an example FACS plot of lung cells from a naïve wild type mouse, the highlighted box shows the presence of CD4^+^ cells. The CD4^+^ T cell KO mice were devoid of CD4^+^ cells (Fig. 1b). This did not impact on the levels of other adaptive immune cell types such as CD8^+^ T cells and B cells (Fig. 1c-d) and tissue, mast cells, eosinophil, neutrophil and alveolar macrophage numbers (Table 2). Genotyping data confirmed the status of the TRPV1 and TRPA1^−/−^ lines (data not shown) and the typical functional phenotype of these lines has been demonstrated in previous publications [26].

**Table 2:** Characterisation of naive CD4^−/−^ mouse line

	Wild type	CD4^−/−^
Eosinophilia(cells/mg of lung tissue) (as % of total white cells)	684 ± 132 (6.3 %)	648 ± 161 (4.3 %)
Neutrophilia(cells/mg of lung tissue) (as % of total white cells)	6443 ± 1048 (45.3 %)	7361 ± 2260 (49.1 %)
Tissue mono/macs(cells/mg of lung tissue) (as % of total white cells)	639 ± 132 (4.5 %)	686 ± 179 (9.4 %)
Alveolar macs(cells/mg of lung tissue) (as % of total white cells)	292 ± 48 (2.1 %)	241 ± 25 (1.6 %)
Mast cells (cells/slide)	66 ± 12	77 ± 10

### Role of CD4^+^ cells in the House Dust Mite driven allergic asthma mouse model

**Fig. 2:**
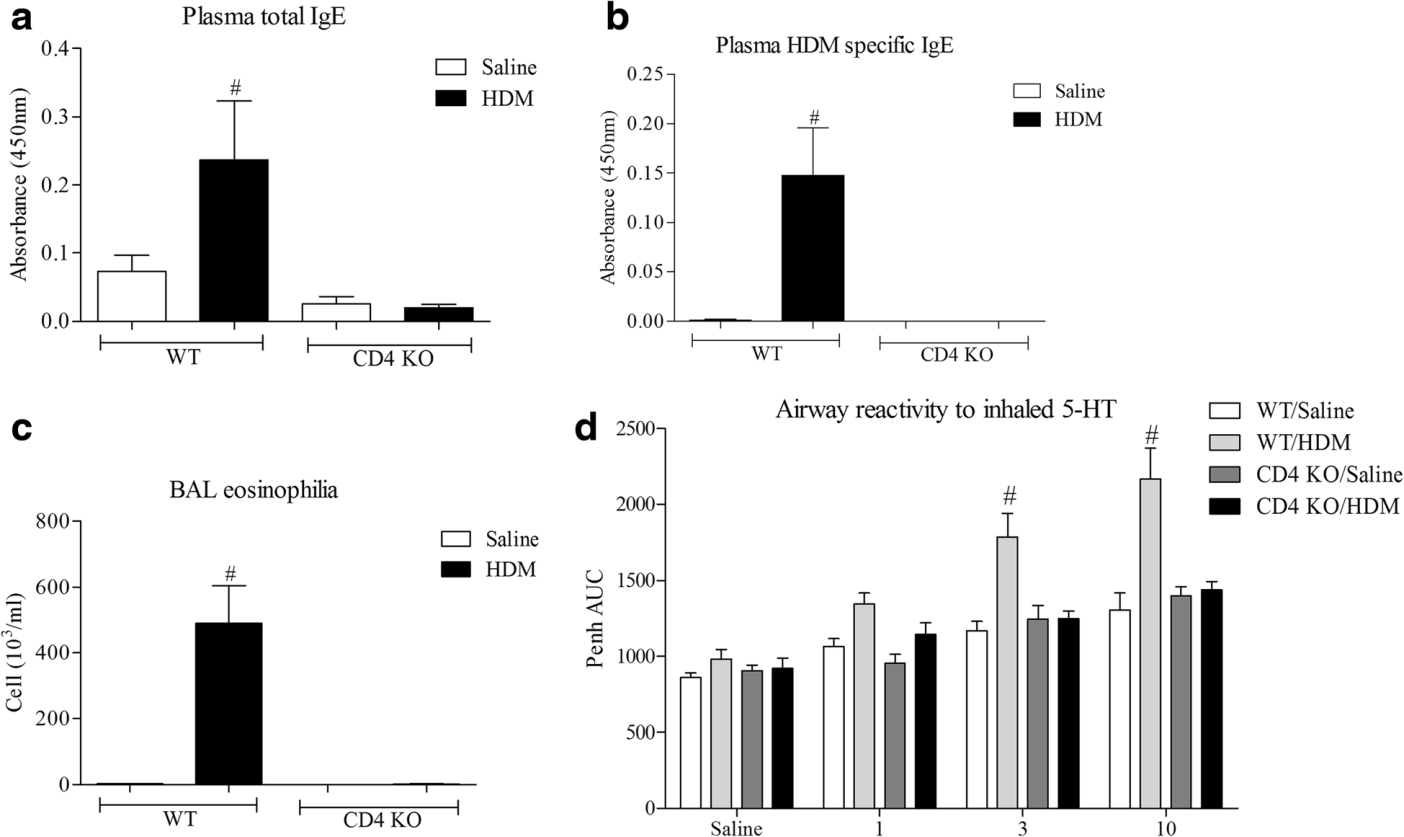
Role of CD4^+^T cells in the House Dust Mite driven allergic asthma mouse model. **a & b**) Total and HDM specific IgE levels in the plasma, respectively, as measured by ELISA. **c** The number of eosinophils in the BAL. **d** Lung function in response to inhaled 5-HT. The percentages of the total white cells for each treatment group are: 3, 66, 0, & 2, respectively, for eosinophils. Data expressed as mean +/− s.e.m. # = *p* < 0.05 using students *T*-test (Mann-Whitney)

Initially, to elucidate the role of CD4^+^ T cells in our murine model of allergic airway disease, WT and CD4^−/−^ mice were sensitised and challenged with HDM. HDM challenge significantly increased serum total/specific IgE (Figure2a and b), BAL eosinophilia (Fig. 2c) and airway reactivity to inhaled 5-HT (Fig. 2d) relative to vehicle challenged controls. CD4^−/−^mice did not display such an augmentation in these parameters, being indistinguishable from vehicle controls (Fig. 2); highlighting the critical function of this cell in the development of the allergic asthma phenotype.

### Role of TRPV1 in the House Dust Mite driven allergic asthma mouse model

**Fig. 3:**
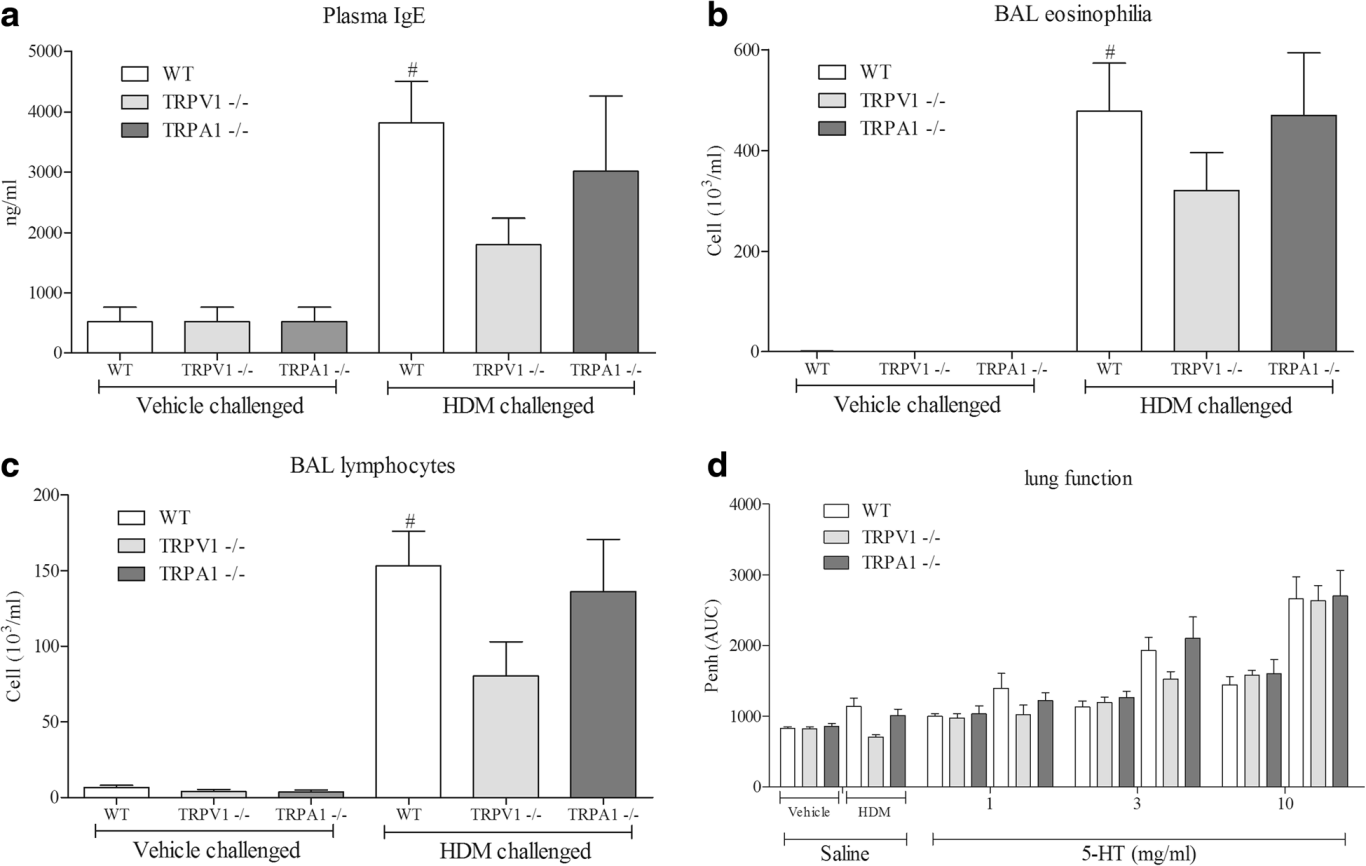
Role of TRPV1/TRPA1 in the mouse model. IgE levels in the plasma as measured by ELISA (**a**), eosinophils (**b**) and lymphocytes (**c**) in the BAL as determined by microscopy using standard morphological criteria in the House Dust Mite driven allergic asthma mouse model in TRPV1 or TRPA1 KO mice compared to wild-type. The percentages of the total white cells for each treatment group are: 0, 0, 0, 65, 61 & 78; 9, 6, 6, 21, 15&23, respectively, for eosinophils and lymphocytes. Lung function to inhaled 5-HT is shown in panel (**d**). Data (*n* = 8) expressed as mean +/− s.e.m. # = *p* < 0.05 using students *T*-test (Mann-Whitney)

Subsequently, we addressed the role of key CD4^+^ T cell regulator, TRPV1, in our murine model of allergic airway disease. As anticipated, HDM challenge significantly increased serum IgE (Fig. 3a), BAL eosinophil (Fig. 3b), lymphocytes (Fig. 3c) and airway reactivity to inhaled 5-HT (Fig. 3d) in wild type mice. TRPV1,but not TRPA1, KOs developed an attenuated allergic asthma phenotype although these effects failed to reach statistical significance (Fig. 3). There was no difference in cellular inflammation or airway reactivity in non-challenged animals (WT vs TRPV1 or TRPA1 KO lines).

### Role of TRPV1 channels in the allergic asthma rat model

**Fig. 4:**
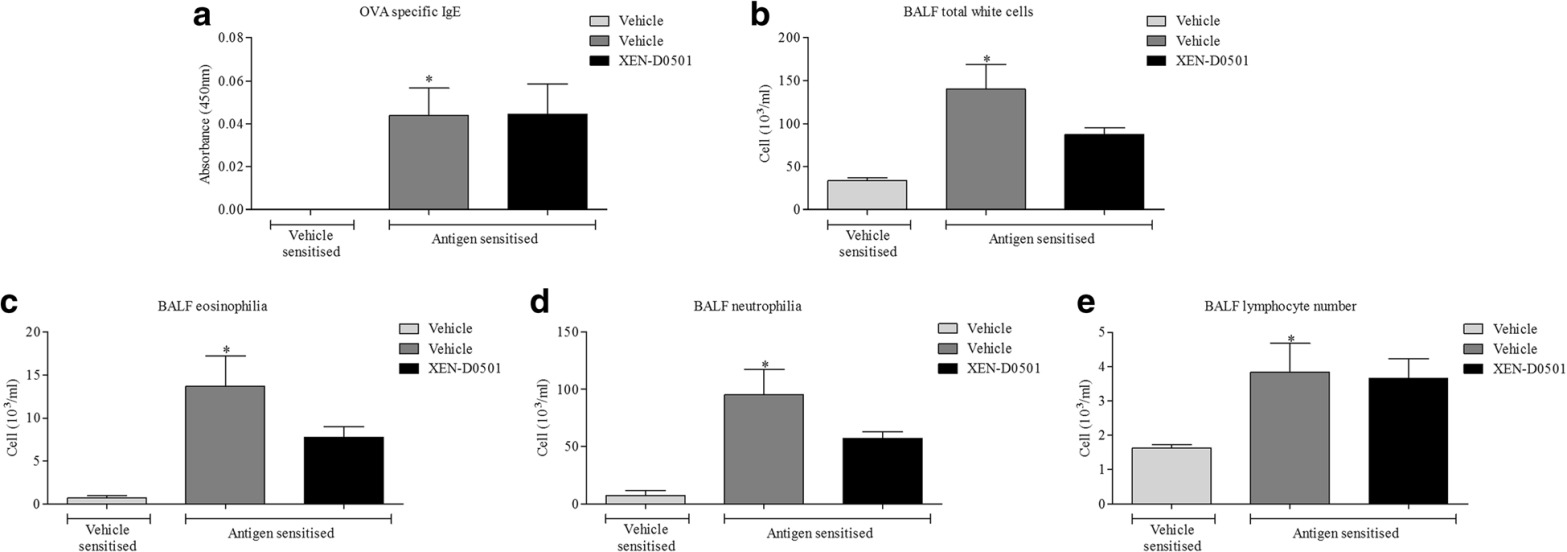
Role of TRPV1 in the rat model. Effect of vehicle (0.5 % MC + 0.2 % tween 80 in saline, 10 ml/kg, i.p., 2 sites; *n* = 8) or TRPV1 blocker (XEN-D0501, 10 mg/kg) 1 h before and 30 min after challenge in the rat allergic asthma model. The following day plasma and BAL fluid was collected. OVA specific IgE levels were measured in the plasma as measured by ELISA (**a**). Total number of white blood cells (**b**), eosinophils (**c**), neutrophils (**d**) and lymphocytes (**e**) in the BAL were determined using microscopy using standard morphological criteria. The percentages of the total white cells for each treatment group are: 2, 10 & 9; 22, 68 & 65; 5, 3 & 4, respectively, for eosinophils, neutrophils and lymphocytes. Data expressed as mean +/− s.e.m. * = *p* < 0.05 using students *T*-test (Mann-Whitney)

**Fig. 5:**
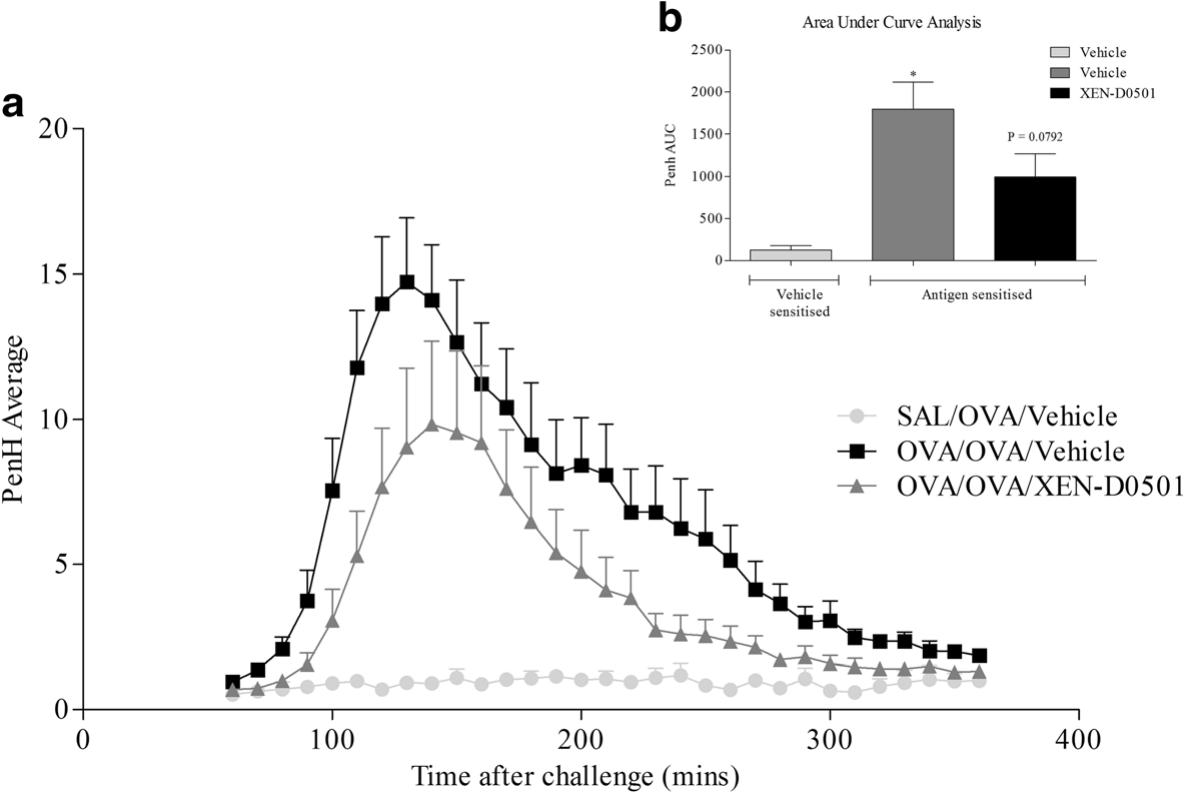
Role of TRPV1 in the rat model. Effect of vehicle (0.5 % MC + 0.2 % tween 80 in saline, 10 ml/kg, i.p., 2 sites; *n* = 8) or TRPV1 blocker (XEN-D0501, 10 mg/kg) 1 h before and 30 min after challenge in the rat allergic asthma model. The late asthmatic response (LAR) was monitored from 1 h after the end of challenge for 6 h. Panel (**a**) shows the raw LAR data and panel (**b**) depicts the mean Area Under the Curve. Data (*n* = 8) expressed as mean +/− s.e.m. * = *p* < 0.05 using students *T*-test (Mann-Whitney)

Given the promising amelioration in pathological phenotype in the TRPV1^−/−^ mice, we investigated the therapeutic potential of manipulating this pathway in a distinct pre-clinical animal model with a disparate allergen. To determine the role of TRPV1 channels in the rat model of allergic asthma we compare responses in vehicle treated rats with those dosed with a selective TRPV1 antagonist, XEN-D0501. The compound was dosed after the animals were sensitised to focus the investigation on the allergic response rather than the development of sensitisation. Antigen (OVA) sensitisation caused a statistically significant increase in plasma OVA specific IgE (Fig. 4a), BALF total cell numbers (Fig. 4b), eosinophils (Fig. 4c), neutrophils (Fig. 4d) and lymphocytes (Fig. 4e) relative to vehicle control. Whilst OVA-specific IgE levels were not impacted on by the TRPV1 inhibitor (Fig. 4a), blockade of the TRPV1 channel resulted in a general reduction in the airway cellular inflammation profile (Fig. 4), although as seen in the mouse model this failed to reach significance. These changes were associated with a reduction in the LAR signal (Fig. 5).

**Fig. 6:**
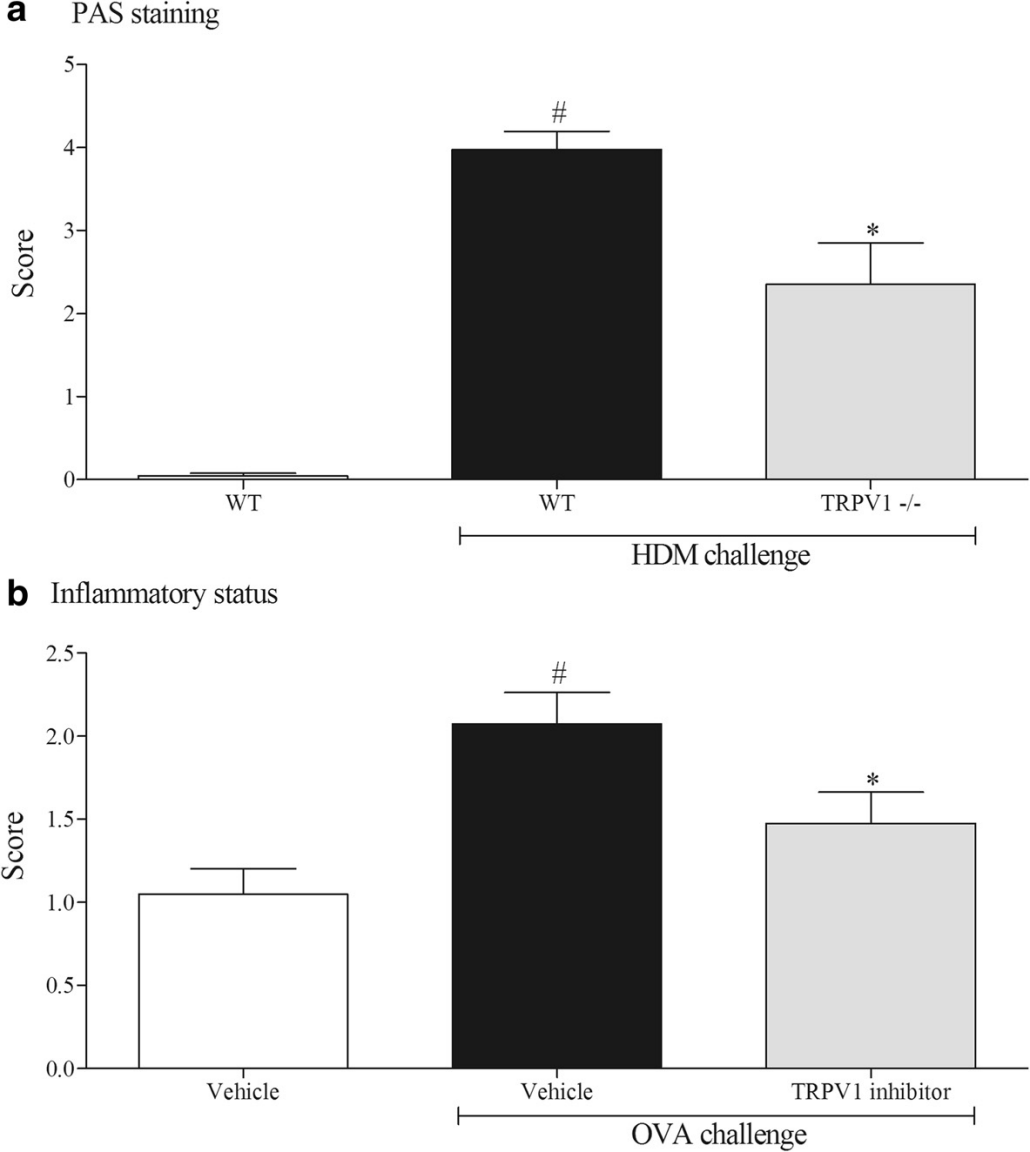
Role of TRPV1 in the mouse and rat model. Histological assessment of lungs from the mouse and rat models. Panel (**a**) represents the level of mucus staining (PAS) in the mouse lungs after HDM challenge. Panel (**b**) shows the inflammatory score of the rat lungs after OVA challenge. Data (*n* = 4-8) expressed as mean +/− s.e.m. # indicates statistical significance between vehicle challenged and antigen challenged (*p* < 0.05 using students *T*-test, Mann-Whitney). * indicates statistical significance when TRPV1 is missing/inhibited, (*p* < 0.05 using students *T*-test, Mann-Whitney)

Histological assessment of the lung tissue from the model systems are shown in Fig. 6. The levels of mucus in the lung tissue were increased in the murine-HDM model (panel A) but not in the rat-OVA model (data not shown). The mice missing functional TRPV1 had reduced levels of mucus compared to the HDM challenged wild type controls (Panel A). In both model systems we measured an increase in the inflammatory score. This was reduced by the TRPV1 inhibitor (Panel B).

## Discussion

CD4^+^ T cells are thought to play a prominent role in the development of allergic asthma, thus targeting them is considered to be a viable means to combat the disease. Recently it has been shown that human and mouse CD4^+^ T cells express the ion channel TRPV1 [7]. Furthermore, modulating TRPV1 can influence activation status, survival and, importantly, the release of mediators thought to be important in the allergic phenotype [5, 6, 27]. Thus we hypothesised that inhibiting the TRPV1 channel would reduce the activity of CD4^+^ T cells thereby modulating the allergic phenotype. To test this hypothesis we used models of allergic asthma in which T cells are known to play a prominent role. We have previously shown that T cells play a dominant role in the OVA-driven Brown Norway rat model which recapitulates an asthma-like phenotype with regard the lung inflammatory response and the LAR (a clinical endpoint that is often used to trial new asthma therapeutics) [20]. To parallel this model system utilising an alternative antigen and a different species, we recently developed a HDM-driven model in the mouse [22]. This model has a number of advantages over the rat model including: the ability to utilise a ‘disease-relevant antigen’, the fact that Alum is not required for the sensitising phase and because it exhibits one of the cardinal characteristics of the clinical asthma phenotype, AHR.

Before profiling the role of TRPV1 in these systems, we confirmed a role for CD4^+^ cells in the HDM-driven model by utilising genetically GM mice that do not possess CD4^+^ T cells. Using this model we compared the allergic phenotype in wild type mice and in mice missing functional TRPV1 (and TRPA1) channels. The data suggests that TRPV1, but not TRPA1, plays a discrete role in several of the functional endpoints assessed in this model system including a reduction in plasma IgE levels, airway cellular inflammation and AHR. In order to confirm a role for TRPV1 in the asthma phenotype we employed an alternative model system/species configured in the Brown Norway rat and in this case utilised a clinically-ready pharmacological inhibitor, XEN-D0501. This enabled us to avoid any possible developmental issues associated with using GM mice and study the effect on the challenge phase, rather than both the sensitisation and challenge phase of the model (which is what is studied in developmental KO mice). Interestingly, while the impact on cellular inflammation is reminiscent of the data in the mouse, the levels of IgE were not altered. This may suggest that TRPV1 plays a role in both the sensitisation and challenge phases; this would parallel the concept of published data demonstrating a role for TRPV1 on T cell function [6, 7]. The reduction in the LAR signal was associated with reduced inflammatory response. In summary, the data suggests that blockade of TRPV1 attenuates the allergic asthma phenotype which is consistent with some previous studies [28–30]. Conversely, others have published that TRPV1 has no role, or can protect against the development of allergic inflammation in the airways [31, 32]. It is not clear why there are these differences; potentially it could be due the different allergens used to provoke the phenotype, the species, whether validation utilised developmental KOs or small molecule inhibitors (and which small molecule inhibitors) and the end-points recorded. However, we have taken into account the differences in experimental settings and have performed one of the most comprehensive studies to date and have obtained a broadly similar picture regardless of the parameters of the model which all points to a discrete role for TRPV1 in the development of the asthma phenotype.

We have suggested a role for TRPV1 in modulation of CD4^+^ T cell function, but cannot rule out a role on other cell types. Indeed, TRPV1 has been reported to be expressed on many cell types in the airway including those thought to play a key role in allergic asthma such as: mast cells, macrophages, epithelial cells, smooth muscle cells, leukocytes and dendritic cells [33–37]. Indeed Rehman et al. have suggested TRPV1 inhibition could be beneficial in attenuating airway epithelial injury and thus reduces asthma features [27]. Furthermore, the TRPV1 receptor is expressed on the peripheral terminals of airway specific, vagal afferent nerves [38] and it has been suggested that the LAR [23] and AHR [30, 39] are reflex events. In addition, activation of this ion channel on sensory afferents has been linked with the release of tachykinins/neuropeptideswhich could be involved in certain functional aspects of the asthma phenotype such as inflammation and bronchoconstriction [40]. It is not clear how the TRPV1 channel is being activated in the model systems. TRPV1 is known to be activated by various pro-inflammatory mediators such as lipoxygenase metabolites which are reported to be increased in the asthmatic airway [8]. Furthermore, the airway of asthmatic patients is thought to be at a lower pH (from 5.2 to 7.1) and the pH is normalised with corticosteroid, treatment [41]. Thus this lower pH could be an endogenous activator of TRPV1 in the diseased airway.

## Conclusion

In conclusion, our data supports the view that targeting TRPV1 could be of clinical benefit in patients with allergic asthma, via a possible role in the modulation of CD4^+^ T cell function. This data, along with other recent publications, informs our thinking on the role of TRP channels in the airways expanding their remit from the control of sensory nerve function to a modulator of key cells involved in adaptive immunity.

## Abbreviations

*AHR* airway hyperresponsiveness, *LAR* late asthmatic response, *TRPV1* transient receptor potential cation channel subfamily V member1, *TRPA1* transient receptor potential cation channel, subfamily A, member 1, *GM* genetically modified, *HDM* house dust mite
